# Improving metagenomic binning results with overlapped bins using assembly graphs

**DOI:** 10.1186/s13015-021-00185-6

**Published:** 2021-05-04

**Authors:** Vijini G. Mallawaarachchi, Anuradha S. Wickramarachchi, Yu Lin

**Affiliations:** School of Computing, College of Engineering and Computer Science, Australian National University, Canberra, Australia

**Keywords:** Metagenomics binning, Contigs, Assembly graphs, Overlapped binning

## Abstract

**Background:**

Metagenomic sequencing allows us to study the structure, diversity and ecology in microbial communities without the necessity of obtaining pure cultures. In many metagenomics studies, the reads obtained from metagenomics sequencing are first assembled into longer contigs and these contigs are then binned into clusters of contigs where contigs in a cluster are expected to come from the same species. As different species may share common sequences in their genomes, one assembled contig may belong to multiple species. However, existing tools for binning contigs only support non-overlapped binning, i.e., each contig is assigned to at most one bin (species).

**Results:**

In this paper, we introduce GraphBin2 which refines the binning results obtained from existing tools and, more importantly, is able to assign contigs to multiple bins. GraphBin2 uses the connectivity and coverage information from assembly graphs to adjust existing binning results on contigs and to infer contigs shared by multiple species. Experimental results on both simulated and real datasets demonstrate that GraphBin2 not only improves binning results of existing tools but also supports to assign contigs to multiple bins.

**Conclusion:**

GraphBin2 incorporates the coverage information into the assembly graph to refine the binning results obtained from existing binning tools. GraphBin2 also enables the detection of contigs that may belong to multiple species. We show that GraphBin2 outperforms its predecessor GraphBin on both simulated and real datasets. GraphBin2 is freely available at https://github.com/Vini2/GraphBin2.

**Supplementary Information:**

The online version contains supplementary material available at 10.1186/s13015-021-00185-6.

## Background

With the advent of high throughput sequencing approaches, the field of metagenomics has enabled us to access and study the genetic material of entire microbial communities [1, 2]. A microbial community is usually a complex mixture of multiple species and recovering these species is crucial to understand the behaviour and functions within such communities. To characterise the composition of a sample, we cluster metagenomic sequences into bins that represent different taxonomic groups such as species, genera or higher levels [3]. This process is known as *metagenomics binning*. Various efforts have been made to bin reads directly (prior to assembly) [4–10]. However, reads are considered as too short to produce accurate and reliable binning results for downstream analysis [11]. Hence, the standard approach followed during metagenomics analysis is to assemble short reads into longer contigs and then cluster these resulting contigs into bins that represent different species, genera, etc [3].

Existing metagenomic contig-binning tools can be divided into two categories. These two categories are (1) reference-based binning and (2) reference-free binning. Reference-based binning approaches [12–15] rely on a database consisting of reference genomes and thus may not be applicable in many metagenomic samples when the reference genomes of novel species are not available. On the contrary, reference-free binning tools use unsupervised approaches to group contigs into unlabelled bins which correspond to different taxonomic groups, solely based on the information obtained from the contigs [3]. These reference-free binning methods become very convenient when analysing environmental samples, especially when many species are not found in currently available reference databases [16]. Most of the reference-free binning tools make use of the composition and/or abundance (coverage) information of the contigs to bin them [17–23]. Even though contigs are assembled from reads using assembly graphs, the majority of the existing binning tools do not make use of the information available in the assembly graph. Recently, GraphBin [24] has been developed to use the connectivity information in the assembly graph to refine the binning results of existing tools because contigs connected to each other in the assembly graph are more likely to belong to the same taxonomic group [25].

Different bacterial genomes in a metagenomic sample may share similar genes and genomic regions [26], which is a major challenge in assembling metagenomic reads into contigs [27]. Therefore, some assembled contigs from metagenomic reads may be shared by multiple species in the sample. However, very few contig-binning tools support overlapped binning (i.e., assigning shared contigs to multiple species). S-GSOM [28] abstracts the flanking sequences of highly conserved 16S rRNA and incorporates them into Growing Self-Organising Maps (GSOM) to bin contigs into overlapping bins. MetaPhase [29] uses Hi-C reads to scaffold assembled contigs into assemblies of individual species and allows certain contigs to belong to multiple species. However, the applications of S-GSOM and MetaPhase are limited due to their required additional sequencing effort (e.g., 16S RNA or Hi-C sequencing). As shared contigs correspond to shared vertices between different genomic paths on the assembly graph [27], it is worth investigating whether it is possible to infer such shared contigs from the assembly graph without additional sequencing requirements.

In this paper, we present GraphBin2, the new generation of GraphBin, to improve binning results using the assembly graph. While GraphBin only uses the topology information of the assembly graph, GraphBin2 improves the algorithms to adjust existing binning results and to support overlapped binning based on both the connectivity and coverage information of assembly graphs. Experimental results show that GraphBin2 not only improves existing binning results, but also infers contigs that may belong to multiple species. Furthermore, we have experimentally shown that GraphBin2 could be applied to long-read assemblies as well.

## Methods

**Fig. 1:**
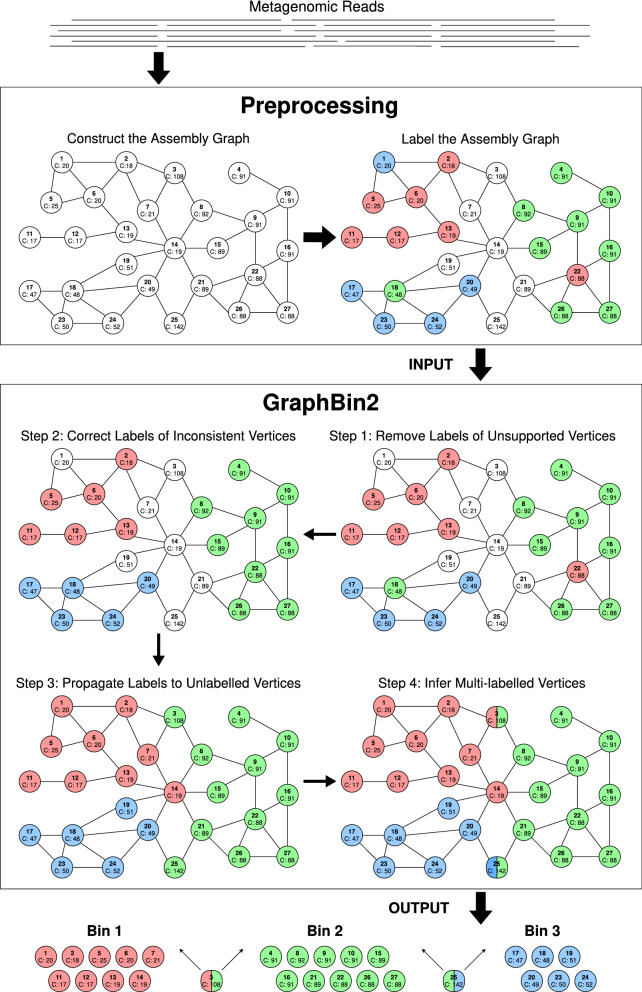
The workflow of GraphBin2. The preprocessing steps of GraphBin2 assemble reads into contigs using the assembly graph and then bin the contigs using existing contig-binning tools. GraphBin2 takes this labelled assembly graph as input, removes unsupported labels, corrects the labels of inconsistent vertices, propagates labels to unlabelled vertices and infers vertices with multiple labels. Finally, GraphBin2 outputs the bins with their corresponding contigs

Figure 1 denotes the workflow of GraphBin2. The preprocessing steps of GraphBin2 assemble reads into contigs using the assembly graph and then bin the contigs (i.e., assign coloured labels to contigs) using existing contig-binning tools. GraphBin2 takes this labelled assembly graph as input, removes unsupported labels, corrects the labels of inconsistent vertices, propagates labels to unlabelled vertices and finally infers vertices with multiple labels (colours).

**Fig. 2:**
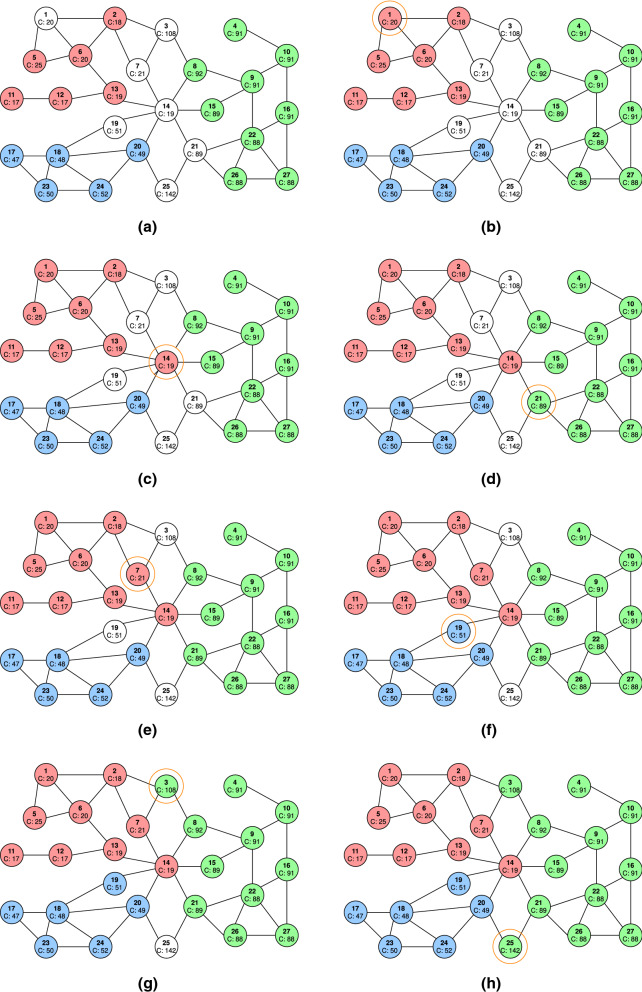
Step-by-step illustration of how labels are propagated in Step 3 of the GraphBin2 Workflow on the assembly graph shown in **a**. The following candidate propagation actions will be executed in the given order. (1) The candidate propagation action (1,0,6,1) is executed. Vertex 1 receives the red label from vertex 6 as shown in **b**. (2)The candidate propagation action (1,0,13,14) is executed. Vertex 14 receives the red label from vertex 13 as shown in **c**. (3) The candidate propagation action (1,1,22,21) is executed. Vertex 21 receives the green label from vertex 22 as shown in **d**. (4) The candidate propagation action (1,2,14,7) is executed. Vertex 7 receives the red label from vertex 14 as shown in **e**. (5) The candidate propagation action (1,3,18,19) is executed. Vertex 19 receives the blue label from vertex 18 as shown in **f**. (6) The candidate propagation action (1,16,8,3) is executed. Vertex 3 receives the green label from vertex 8 as shown in **g**. (7) The candidate propagation action (1,53,21,25) is executed. Vertex 25 receives the green label from vertex 21 as shown in **h**

### Preprocessing

In this step, we assemble the next generation reads (e.g., Illumina reads with length ranging from 75 to 300 bp) into contigs using the assembly graph. There are two dominant paradigms for genome assembly: overlap-layout-consensus (or string graphs) [30] and de Bruijn graphs [31]. We select one representative assembler from each paradigm, SGA [32] and metaSPAdes [27] respectively, to demonstrate the adaptability of GraphBin2. In order to show that GraphBin2 could be in principle applied to long-read assemblies, we also considered a simulated dataset which was assembled using metaFlye [33], a popular metagenomics long-read assembler.

In the assembly graph, each vertex represents a contig with *coverage* denoting the average number of reads that map to each base of the contig and each edge indicates a significant overlap between a pair of contigs. In an ideal case, a genome corresponds to a path in the assembly graph and its genomic sequence corresponds to the concatenation of contigs along this path. Hence, if two contigs are connected by an edge in the assembly graph, they are more likely to belong to the same genome. Previous studies [24, 25] have shown that the connectivity information between contigs can be used to refine and improve binning results. In the assembly graph of metagenomic datasets, different genomes usually correspond to different paths in the assembly graph. If two genomes share a common contig (e.g., unresolved “interspecies repeat” [27]), the corresponding vertex would be shared by two genomic paths in the assembly graph.

After assembling reads into contigs using assembly graphs, GraphBin2 uses an existing contig-binning tool to derive an initial binning result. Note that most of the existing tools for binning contigs require a minimum length for the contigs (e.g., 1000 bp for MaxBin2 [23] and SolidBin [22], 500 bp for BusyBee Web [16] and 1500 bp for MetaBAT2 [19]). Therefore, many short contigs in the assembly graph will be discarded, resulting in low recall values as a common limitation of existing binning tools. For example, 65% of the contigs in the metaSPAdes assembly of the Sharon-All dataset were discarded by MaxBin2 due to their short length.

### Step 1: Remove labels of unsupported vertices

A linear (or circular) chromosome usually corresponds to a path (or a cycle) that traverses multiple vertices in the assembly graph. If two contigs belong to the same chromosome, they are likely to be connected by a path which consists of other contigs from the same chromosome. Therefore, a labelled vertex is defined as *supported* if and only if one of the following conditions hold:

- It is an isolated vertex
- It directly connects to a vertex of the same label
- It connects to a vertex of the same label through a path that consists of only unlabelled vertices.

Otherwise, a labelled vertex is defined as *unsupported*. Note that the definition of *unsupported* vertices in GraphBin2 is more strict than *ambiguous* vertices in GraphBin.^1^ For example, in the initial labelled assembly graph of Fig. 1, vertex 2 in red is supported by vertex 6 in red as they are directly connected. Note that vertex 18 in green is also supported by vertex 15 in green as there exists a path (i.e., $18 \rightarrow 19 \rightarrow 14 \rightarrow 15$) between them that traverses only unlabelled vertices (i.e., 19 and 14). However, vertex 1 in blue is unsupported as it cannot reach another blue vertex through a path consisting of only unlabelled (white coloured) vertices.

To check whether a labelled vertex is *supported* or *unsupported*, a naive approach is to perform a breadth-first-search from each labelled vertex. A refined algorithm first initialises all labelled vertices as unsupported and scans the graph to identify all labelled vertices that are either isolated or directly connected to a vertex of the same label and classifies them as supported vertices. This refined algorithm then uses breadth-first-search to find all connected components that consist of only unlabelled vertices and for each component *Component* stores a set of labelled vertices *N*(*Component*) that are connected to vertices in *Component*. If multiple labelled vertices in *N*(*Component*) have the same label, these vertices are supported because they connect to each other through a path that consists of only unlabelled vertices in *Component*. GraphBin2 removes the labels for all unsupported vertices because these labels may not be reliable. For example, the label of the unsupported vertex 1 is removed by GraphBin2 in Step 1 of Fig. 1.

### Step 2: Correct labels of inconsistent vertices

After Step 1, each non-isolated labelled vertex *v* is supported by at least one vertex with the same label. The closer two vertices are in the assembly graph, the more likely they have the same label. For each vertex *v*, we introduce a *labelled score*, *S*(*v*, *x*), for each label *x* by considering all vertices of label *x* that are directly connected to *v* or connected to *v* through a path that consists of only unlabelled vertices. A vertex *t* of label *x* contributes to *S*(*v*, *x*) by $2^{-D(v,t)}$ where *D*(*v*, *t*) is the shortest distance between *v* and *t* using only unlabelled vertices. This distance is measured by the number of edges in a path and $D(v,t)=1$ if *v* and *t* are directly connected. Therefore, the *labelled score* *S*(*v*, *x*) is the sum of contributions from all vertices of label *x* that are directly connected to *v* or connected to *v* through a path that consists of only unlabelled vertices. In Step 1 of Fig. 1, vertex 17 contributes 1/2 to *S*(18, *blue*) because $D(17,18)=1$ and vertex 8 contributes 1/8 to *S*(18, *green*) because $D(8,17)=3$. The labelled score of *S*(18, *blue*) is 2 to which all four blue vertices 17, 20, 23 and 24 contribute 1/2 respectively while $S(18, green)=5/16$ to which vertex 8 contributes 1/8, vertex 15 contributes 1/8 and vertex 26 contributes 1/16.

A labelled vertex *v* of label *x* is defined as *inconsistent* if and only if the *labelled score* of its current label *x* times $\alpha$ is less than or equal to the *labelled score* of another label *y* where $\alpha$ is a parameter, i.e., $\alpha \times S(v,x) \leqslant S(v,y)$. We have set $\alpha =1.5$ in the default settings of GraphBin2. In Step 1 of Fig. 1, vertex 18 in green is an inconsistent vertex because $1.5 \times S(18, green)= 1.5 \times 5/16 = 0.47$ is less than $S(18, blue)=2$.

Again, GraphBin2 uses the breadth-first-search to check if a labelled vertex is *inconsistent*. GraphBin2 corrects the label of an inconsistent vertex *v* to another label that maximises the labelled score. For example, GraphBin2 corrects the label of vertex 18 from green to blue and corrects the label of vertex 22 from red to green (refer from Step 1 to Step 2 in Fig. 1).

### Step 3: Propagate labels to unlabelled vertices

As existing contig-binning tools discard contigs due to their short lengths in the initial binning, many vertices are still unlabelled in the current assembly graph. In this step, we will propagate existing labels to the remaining unlabelled vertices using the assembly graph. There are two intuitions behind this label propagation process. Firstly, vertices that are closer to each other in the assembly graph are more likely to have the same label. Secondly, vertices with similar coverages are more likely to have the same label because contigs from the same genome usually have similar coverages [18, 34]. GraphBin2 uses both the connectivity and coverage information of the assembly graph to propagate the labels.

For each unlabelled vertex *v* with coverage *c*(*v*) (i.e., coverage of the contig that corresponds to the vertex), a candidate propagation action $(D(v,t),|c(v)-c(t)|, t, v)$ is recorded as a tuple where *t* is the nearest labelled vertex to *v*, *c*(*t*) is the coverage of *t* and *D*(*v*, *t*) is the shortest distance between *v* and *t* (as defined in Step 2). Given two candidate propagation actions, $(d_1,c_1,t_1,v_1)$ and $(d_2,c_2,t_2,v_2)$, GraphBin2 will execute $(d_1,c_1,t_1,v_1)$ before $(d_2,c_2,t_2,v_2)$, i.e., propagating the label of $t_1$ to $v_1$ before propagating the label of $t_2$ to $v_2$, if ($d_1 < d_2$) or ($c_1 < c_2$ and $d_1 = d_2$). In other words, GraphBin2 puts more emphasis on the connectivity information than the coverage information because the edges in the assembly graph are expected to be more reliable than the coverage information on vertices, especially for vertices corresponding to short contigs (which are discarded by initial binning tools).

GraphBin2 first uses the breadth-first-search to compute all candidate propagation actions for unlabelled vertices and sort them into a ranked list according to the order defined above. At each iteration, GraphBin2 executes the first candidate propagation action and then updates the ranked list of candidate propagation actions. Note that one unlabelled vertex receives its label at each iteration and updating the ranked list of candidate propagation actions can be done efficiently by breadth-first-search from this unlabelled vertex.

Figure 2 shows how GraphBin2 propagates labels from Step 2 to Step 3 in Fig. 1. Figure 2a denotes the assembly graph after correcting labels of inconsistent vertices (after Step 2). The step-by-step label propagation process is explained in the remaining figures in Fig. 2.

Note that this label propagation process in GraphBin2 improves on the label propagation algorithm in GraphBin by incorporating both the connectivity and coverage information in the assembly graph. So far, GraphBin2 does not generate multi-labelled vertices. In the next step, we will show how GraphBin2 uses the labelling, connectivity and coverage information together on the assembly graph to infer multi-labelled vertices.

### Step 4: Infer multi-labelled vertices

Contigs belonging to multiple genomes correspond to multi-labelled vertices in the assembly graph. What are the characteristics of shared contigs between multiple species? Firstly, a contig shared by multiple genomes may connect other contigs in these genomes. Secondly, the coverage of a contig shared by multiple genomes should be equal to the sum of coverages of these genomes in the ideal case. After label propagation, vertices of the same label are likely to form connected components in the assembly graph and multi-labelled vertices are likely to be located along the borders between multiple connected components where distinct labels meet and have a coverage similar to the sum of the average coverages of multiple components that they belong to.

GraphBin2 checks labelled vertices that are connected to vertices of multiple different labels. The average coverage of a connected component *P* is calculated by $\frac{\sum {}{} c(i) \times L(i)}{\sum {}{} L(i)}$ for each vertex *i* in the connected component *P*, where *c*(*i*) is the coverage of the vertex *i* and *L*(*i*) is the length of the contig corresponding to vertex *i*. Assume *v* is a labelled vertex *v* from a component *P*, the coverage of *v* is *c*(*v*) and the average coverage of *P* is *c*(*P*). When *c*(*v*) is larger than *c*(*P*) and *v* is connected to other components $P_1,P_2,\ldots ,P_k$ with different labels, it is possible that *v* also belongs to one or more components (in addition to *P*). For example, if *v* belongs to *P*, $P_i$ and $P_j$ in the ground-truth, the coverage of *v*, *c*(*v*), is expected to be close to the sum of average coverages of the above three components, $c(P)+c(P_i)+c(P_j)$. In fact, finding which components in $\{P_1,P_2,\ldots ,P_k\}$ that *v* also belongs to (in addition to *P*) can be modelled as the following subset sum problem [35]. Given a set of positive numbers $\{c(P_1),c(P_2),\ldots ,c(P_k)\}$, find a subset whose sum is or is closest to $c(v)-c(P)$. Then *v* will be assigned to the corresponding components in this subset as well as to *P*. Note that it is possible that the selected subset is empty and thus *v* only belongs to *P*.

In all of our experiments, the maximum number of different components that a vertex connects to in the assembly graph is less than 5. We use a brute-force way to enumerate all possible combinations of components and find out the combinations that best explain the observed coverages. For example, after Step 3 in Fig. 1, vertex 3 in green connects to another red component. The coverage of vertex 3 is 108 while the average coverage of the green component is 95 and the average coverage of the red components is 19. Because the coverage of vertex 3 (108) is closer to the sum of average coverages of green and red components (95+19=114) compared to the average coverage of the green component (95), vertex 3 is assigned both green and red labels. Similarly, the coverage of vertex 25 (142) is closer to the sum of average coverages of green and blue components (95 + 49 = 144) compared to the average coverage of the green component (95). Hence, vertex 25 is assigned both green and blue labels. In the same assembly graph after Step 3 in Fig. 1, vertex 14 in red does not gain any other labels because its own coverage is closest to the average coverage of the red component (19) compared to other possible combinations (i.e., red+blue, red+green, green+blue and red+green+blue).

## Experimental setup

### Datasets

#### Simulated datasets

We simulated three metagenomic datasets according to the species found in the *simMC+* dataset [23]. These datasets were simulated each containing 5 species (referred as Sim-5G), 10 species (referred as Sim-10G) and 20 species (referred as Sim-20G) respectively. Paired-end reads were simulated using the tool InSilicoSeq [36] modelling a MiSeq instrument with 300 bp mean read length.

To benchmark the performance of GraphBin2 on complex metagenomic datasets, we simulated a dataset with the 50 most abundant species found in the *simMC+* dataset [23]. This dataset consisting of MiSeq reads is referred as 50G-SR. Moreover, we used the *100-genomes* long-read dataset [37] which consisted of simulated PacBio reads of 100 species to evaluate the performance of GraphBin2 on long-read assemblies. This dataset has been simulated by the long-read simulator SimLoRD [38] using default parameters for PacBio reads. We refer to this dataset as 100G-LR. Further details about the simulated datasets can be found in Section 1 of Additional file 1.

#### Real datasets

We used the preborn infant gut metagenome, commonly known as the Sharon dataset [39] (NCBI accession number *SRA052203*). There are 18 Illumina (Illumina HiSeq 2000) runs available for this dataset. One run *SRR492184* is included as a representative dataset (referred as Sharon-1) and all the 18 Illumina runs are combined to form the Sharon-All dataset in our experiments.

We also used the Lake Biwa bacterioplankton metagenome dataset ([40]) which consists of bacterioplankton obtained from the Lake Biwa, Japan (NCBI BioProject number *PRJDB6644*, run *DRR125127*, referred as Lake Water) and consists of Illumina MiSeq paired-end reads.

Further details on the Sharon and Lake Water datasets can be found in Section 1 of the Additional file 1.

### Tools used

To derive the assembly graph from short reads, there are two dominant assembly paradigms, de Bruijn graphs [31] and overlap-overlap-layout-consensus (or string graphs) [30]. We selected one representative tool from each paradigm to show the effectiveness of GraphBin2. To represent the de Bruijn graph paradigm, we used metaSPAdes [27] (from SPAdes version 3.13.0 [41]) with its default parameters to generate the assembly graph. As for the overlap-layout-consensus paradigm, we selected SGA (version 0.10.15) [32] to derive the assembly graph. We used the long-read metagenomic assembler metaFlye [33] (available in Flye version 2.4.2 [42]) with its default parameters to assemble the 100G-LR dataset.

We used CONCOCT (version 1.1.0) [17] and MaxBin2 (version 2.2.5) [23] with default parameters, and SolidBin (version 1.3) [22] in SolidBin-SFSmode to obtain the initial binning results for our experiments. CONCOCT, MaxBin2 and SolidBin are considered as hybrid contig-binning tools as they use both the composition and coverage information. They make use of tetranucleotide frequencies and coverages of reads with different machine learning approaches to bin contigs. Note that CONCOCT, MaxBin2 and SolidBin only bin contigs which are longer than 1000 bp by default. We also compared GraphBin2 with its predecessor GraphBin [24]. The commands used to run all the assembly and binning tools can be found in Section 2 of Additional file 1.

### Evaluation criteria

Since the reference genomes of the simulated datasets were known, we used BWA-MEM [43] to align the contigs to their reference genomes to determine the ground truth species to which the contigs actually belonged to. For each contig, the alignment lengths for each species were recorded. A contig is considered to belong to one species if the longest alignment to this species covers at least 50% of the contig length. Furthermore, isolated contigs (corresponding vertices with zero degree in the assembly graph) were not considered for the ground-truth set of the datasets.

For the Sharon dataset, we considered the annotated contigs from 12 species which are available at https://ggkbase.berkeley.edu/carrol/organisms as references. For the Lake Water dataset, we considered the assembled genomes provided by the authors as ground truth species. A process similar to the simulated datasets was followed for the Sharon and Lake Water datasets to determine the origin species of contigs and alignment lengths to species.

To evaluate the binning results of CONCOCT [17], MaxBin2 [23], SolidBin [22], GraphBin [24] and GraphBin2, we used the metrics (1) precision, (2) recall and (3) F1-score which have been used in previous studies [17, 24, 44]. The binning result is denoted as a $K\times S$ matrix where *K* is the number of bins identified by the binning tool and *S* is the number of species available in the ground truth. In this matrix, the element $a_{ks}$ denotes the number of contigs binned to the $k^{th}$ bin and belongs to the $s^{th}$ species. *Unclassified* denotes the number of contigs that are unclassified or discarded by the tool. Following are the definitions and equations that were used to calculate the precision, recall and F1-score.

1 $$\begin{aligned} Precision= & {} \frac{\sum _{k}max_s \{a_{ks}\}}{\sum _{k}\sum _{s}a_{ks}} \end{aligned}$$

2 $$\begin{aligned} Recall= & {} \frac{\sum _{s}max_k \{a_{ks}\}}{(\sum _{k}\sum _{s}a_{ks}+Unclassified)} \end{aligned}$$

3 $$\begin{aligned} F1= & {} 2\times \frac{Precision\times Recall}{Precision+Recall} \end{aligned}$$

To evaluate whether a vertex in the assembly graph corresponds to a contig that may belong to multiple species, we align this contig to genomes of ground-truth species and record the best alignment against each species, respectively. Then we introduce a parameter $Ratio_{(2^{nd}/1^{st})}$ as the ratio between the alignment lengths of the second longest alignment and the longest alignment. If a contig is aligned to only one species (i.e., there is no alignment to another species), then $Ratio_{(2^{nd}/1^{st})}=0$. If a contig is aligned to multiple species, the higher the $Ratio_{(2^{nd}/1^{st})}$ is, the more likely that this contig belongs to multiple species. The violin plots of $Ratio_{(2^{nd}/1^{st})}$ are computed for both inferred multi-labelled and single-labelled contigs in the next section to demonstrate how $Ratio_{(2^{nd}/1^{st})}$ varies for each type of contigs.

## Results and discussion

### Binning results

**Fig. 3:**
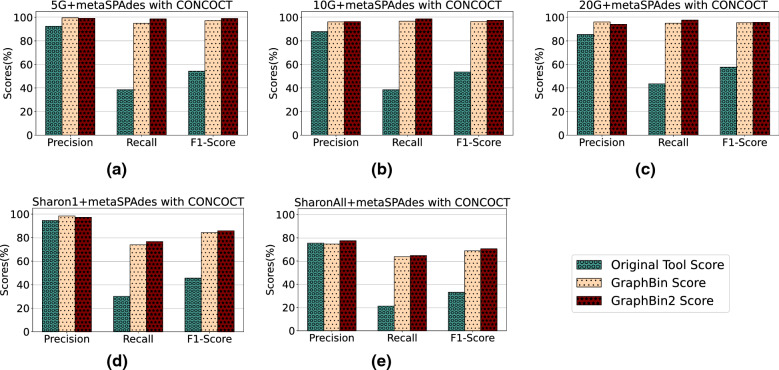
Comparison of binning results of CONCOCT [17], GraphBin [24] and GraphBin2 (on top of CONCOCT results) using assembly graphs built by metaSPAdes [27]

**Fig. 4:**
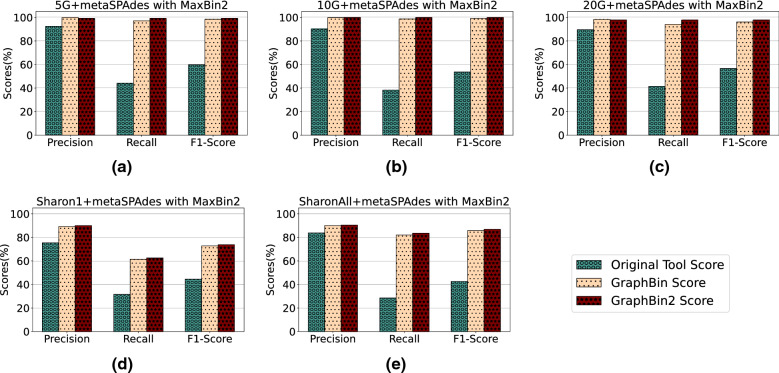
Comparison of binning results of MaxBin2 [23], GraphBin [24] and GraphBin2 (on top of MaxBin2 results) using assembly graphs built by metaSPAdes [27]

**Fig. 5:**
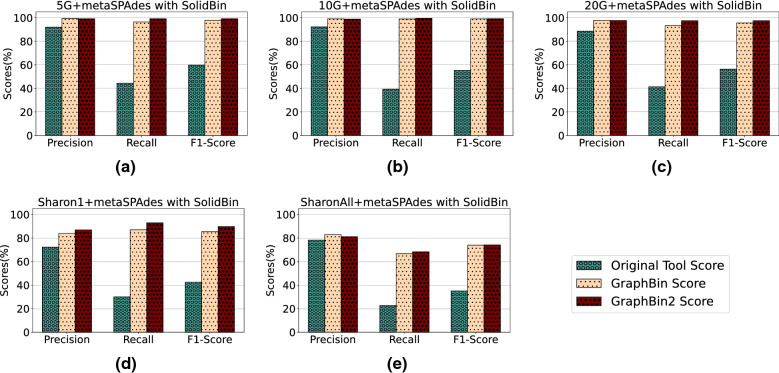
Comparison of binning results of SolidBin [22], GraphBin [24] and GraphBin2 (on top of SolidBin results) using assembly graphs built by metaSPAdes [27]

**Fig. 6:**
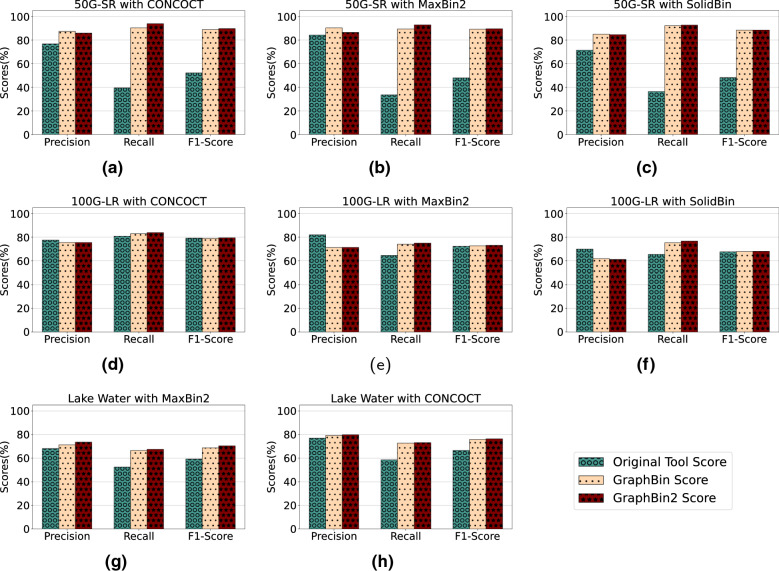
Comparison of binning results of CONCOCT [17], MaxBin2 [23], SolidBin [22], GraphBin [24] and GraphBin2 for the complex datasets 50G-SR, 100G-LR and Lake Water

Figures 3, 4 and 5 demonstrate the results of CONCOCT [17], MaxBin2 [23] and SolidBin [22], respectively with GraphBin [24] and GraphBin2 on top of the initial binning results for the metaSPAdes assemblies. Figure 6 denotes the binning results of all the tools for the complex datasets 50G-SR, Lake Water and 100G-LR. The number of bins identified by the binning tools for each dataset can be found in Table 1. Binning results of the SGA assemblies can be found in Section 3 of Additional file 1.

**Table 1:** The number of bins identified by the binning tools for each dataset

Dataset	Ground truth bins	Assembly type	Binning tool	Number of bins identified
Sim-5G	5	metaSPAdes	CONCOCT	7
			MaxBin2	5
			SolidBin	5
		SGA	CONCOCT	11
			MaxBin2	5
			SolidBin	5
Sim-10G	10	metaSPAdes	CONCOCT	12
			MaxBin2	10
			SolidBin	10
		SGA	CONCOCT	14
			MaxBin2	9
			SolidBin	9
Sim-20G	20	metaSPAdes	CONCOCT	22
			MaxBin2	21
			SolidBin	20
		SGA	CONCOCT	28
			MaxBin2	20
			SolidBin	19
Sharon-1 [39]	12	metaSPAdes	CONCOCT	27
			MaxBin2	5
			SolidBin	5
		SGA	CONCOCT	25
			MaxBin2	5
			SolidBin	4
Sharon-All [39]	12	metaSPAdes	CONCOCT	48
			MaxBin2	11
			SolidBin	9
		SGA	CONCOCT	27
			MaxBin2	8
			SolidBin	5
50G-SR	50	metaSPAdes	CONCOCT	44
			MaxBin2	44
			SolidBin	45
Lake Water [40]	57	metaSPAdes	CONCOCT	149
			MaxBin2	57
			SolidBin	N/A$^{*}$
100G-LR [37]	100	metaFlye	CONCOCT	76
			MaxBin2	76
			SolidBin	86

$^{*}$ SolidBin [22] could not be run on the Lake Water dataset due to insufficient memory

The binning results show that GraphBin2 achieves the best performance in most of the scenarios. The improvement over GraphBin is because GraphBin2 makes use of coverage information additionally, rather than relying only on the graph topology as GraphBin does. Both GraphBin and GraphBin2 have shown significant improvements on recall compared to CONCOCT, MaxBin2 and SolidBin. While CONCOCT, MaxBin2 and SolidBin filter contigs with length shorter than 1000 bp, GraphBin and GraphBin2 are able to bin short contigs using assembly graphs. In a few scenarios, GraphBin2 improved on the recall with a bit of a compromise on the precision compared to GraphBin because GraphBin removes ambiguous labels in the final step. Furthermore, the existence of weak edges (i.e., edges that are not well supported from the data) can form false connections between contigs and can mislead the label propagation process.

### Multi-Labelled Inference Results

**Table 2:** The number of multi-labelled contigs identified by GraphBin2 for the metaSPAdes assemblies and assemblies of the complex datasets using the initial binning result of each binning tool

Dataset	With CONCOCT result	With MaxBin2 result	With SolidBin result
Sim-5G	3	4	5
Sim-10G	6	7	7
Sim-20G	5	11	10
Sharon1 [39]	3	3	2
SharonAll [39]	69	38	30
50G-SR	89	74	74
Lake Water [40]	178	329	N/A$^{*}$
100G-LR [37]	17	10	10

$^{*}$ SolidBin [22] could not be run on the Lake Water dataset due to insufficient memory

**Fig. 7:**
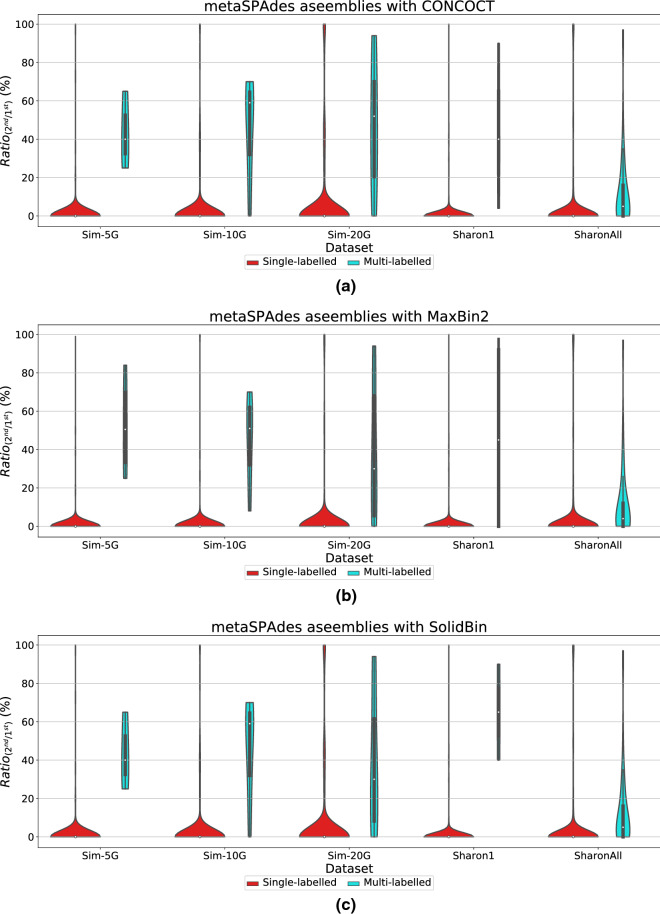
Violin plots for the ratio between the alignment lengths of the second longest alignment and the longest alignment of the single and multi-labelled inference results using GraphBin2 on top of **a** CONCOCT [17], **b** MaxBin2 [23] and **c** SolidBin [22] results for the metaSPAdes assemblies

**Fig. 8:**
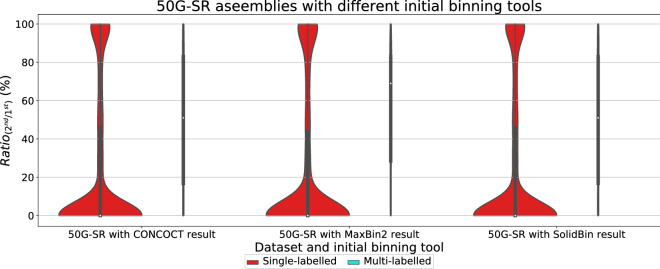
Violin plots for the ratio between the second longest alignment and the longest alignment of the single and multi-labelled inference results using GraphBin2 on top of the initial binning results from CONCOCT [17], MaxBin2 [23] and SolidBin [22] for the 50G-SR assembly

**Fig. 9:**
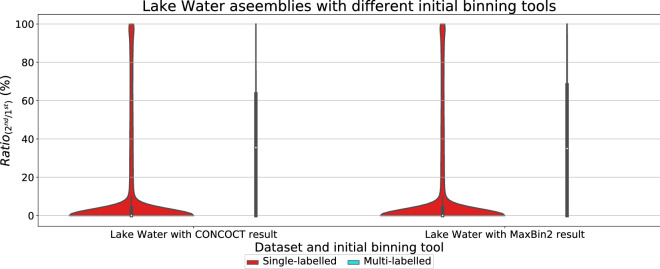
Violin plots for the ratio between the alignment lengths of the second longest alignment and the longest alignment of the single and multi-labelled inference results using GraphBin2 on top of the initial binning results from CONCOCT [17] and MaxBin2 [23] for the Lake Water assembly. $^{*}$ SolidBin [22] could not be run on the Lake Water dataset due to insufficient memory

**Fig. 10:**
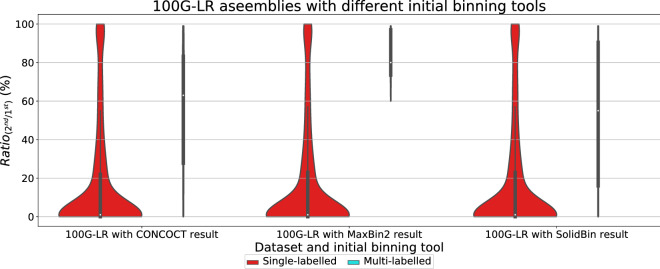
Violin plots for the ratio between the alignment lengths of the second longest alignment and the longest alignment of the single and multi-labelled inference results using GraphBin2 on top of the initial binning results from CONCOCT [17], MaxBin2 [23] and SolidBin [22] for the 100G-LR assembly

One key novelty of GraphBin2 is the introduction of the multi-labelled inference for contigs where GraphBin2 detects possible contigs that may belong to multiple species. Table 2 denotes the number of multi-labelled contigs identified by GraphBin2 for the metaSPAdes assemblies and assemblies of the complex datasets using the initial binning result of the binning tools CONCOCT [17], MaxBin2 [23] and SolidBin [22]. Moreover, for each combination of dataset and initial binning tool, we calculated the ratio $Ratio_{(2^{nd}/1^{st})}$ (please refer to section “Evaluation criteria”) of single and multi-labelled contigs produced by GraphBin2. Then we plotted the violin plots of $Ratio_{(2^{nd}/1^{st})}$ in Figs. 7, 8, 9 and 10 to demonstrate how $Ratio_{(2^{nd}/1^{st})}$ varies for different datasets. Multi-labelled inference results of the SGA assemblies can be found in Section 3 of Additional file 1.

According to Figs. 7, 8, 9 and 10, the multi-labelled contigs identified by GraphBin2 for most of the datasets have a high mean value (much greater than zero) for $Ratio_{(2^{nd}/1^{st})}$, suggesting that these identified contigs have significant alignments to multiple species. Moreover, the mean value of $Ratio_{(2^{nd}/1^{st})}$ for the single-labelled contigs identified by GraphBin2 is close to zero, suggesting that the majority of the contigs only belong to one species. The clear distinction between the $Ratio_{(2^{nd}/1^{st})}$ of inferred single and multi-labelled contigs in these datasets demonstrates the effective detection of contigs that may belong to multiple species by GraphBin2. Note that the relatively low mean value of $Ratio_{(2^{nd}/1^{st})}$ for the Sharon-All dataset can be due to repeats and weak edges in complex assembly graphs, i.e., contigs that represent repeats within one species tend to have higher coverage and may be misinterpreted as multi-labelled contigs if there exist weak edges connecting them to contigs in other species. The possible multi-labelled contigs in the 50G-SR and 100G-LR datasets which are not identified by GraphBin2 may be due to the underestimation of the number of bins, misassemblies and fragmentation of the assembly graphs, especially for datasets with a large number of species.

### Visualisation of the assembly graph

**Fig. 11:**
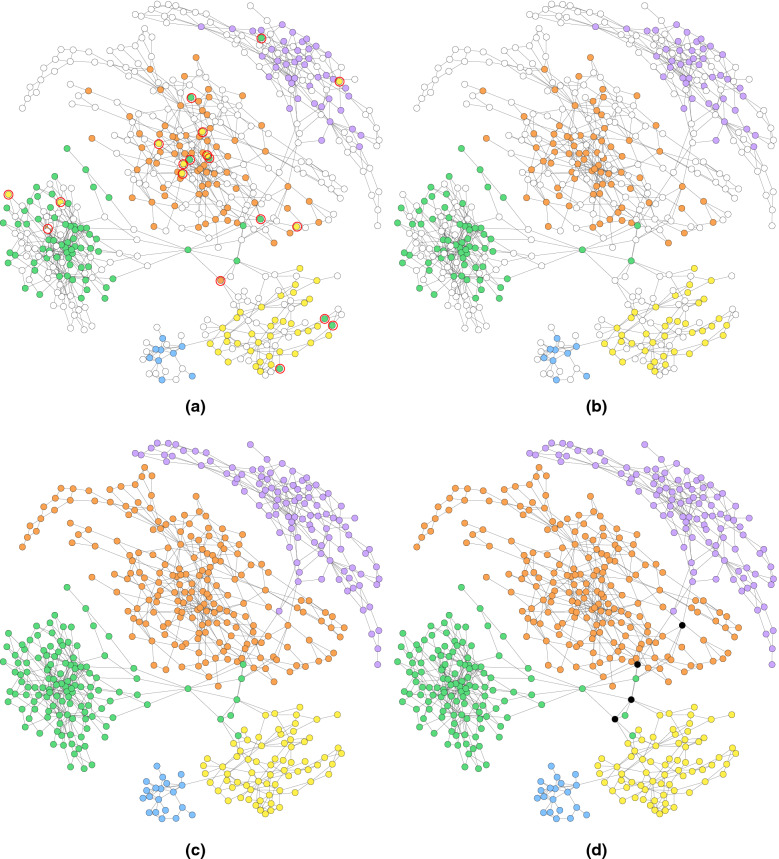
The labelling of the assembly graph of Sim-5G dataset based on **a** the initial MaxBin2 result (un-binned contigs are denoted by white coloured vertices and mis-binned contigs are circled in red), **b** after removing labels of unsupported vertices and correcting labels of inconsistent vertices, **c** after propagating labels of unlabelled vertices, **d** after determining multi-labelled vertices (black coloured vertices) by GraphBin2

**Fig. 12:**
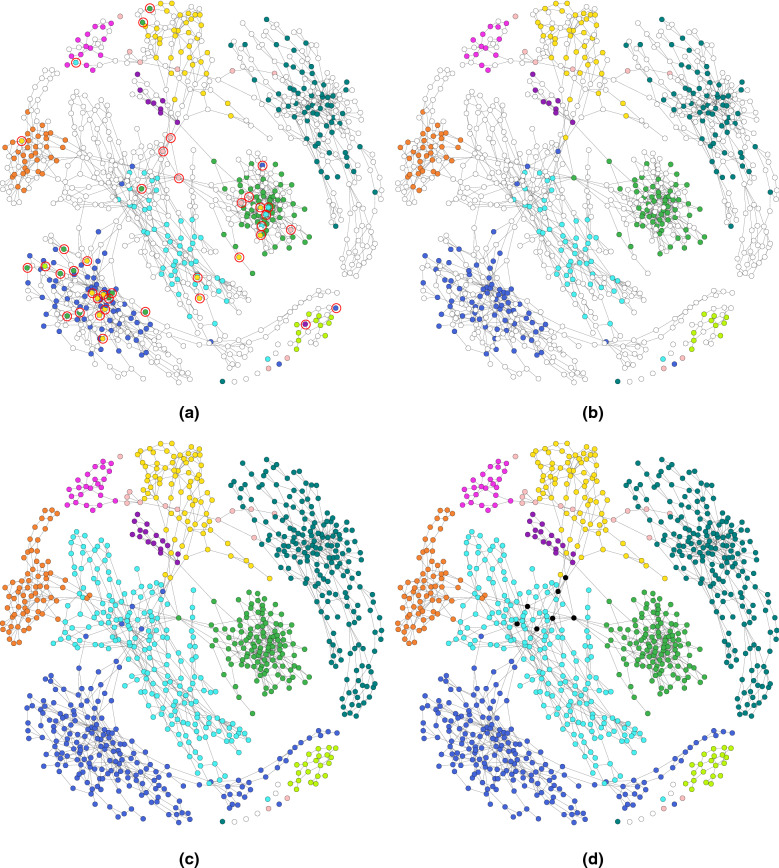
The labelling of the assembly graph of Sim-10G dataset based on **a** the initial MaxBin2 result (un-binned contigs are denoted by white coloured vertices and mis-binned contigs are circled in red), **b** after removing labels of unsupported vertices and correcting labels of inconsistent vertices, **c** after propagating labels of unlabelled vertices, **d** after determining multi-labelled vertices (black coloured vertices) by GraphBin2

Figures 11 and 12 denote the labelling of the contigs in the metaSPAdes assembly graphs of the Sim-5G and Sim-10G datasets at different stages as it undergoes the processing of GraphBin2. White coloured vertices denote un-binned contigs and the rest of the coloured vertices denote the labelled contigs. In Figs. 11a and 12a, we can see that some mis-binned contigs are identified (circled in red) as differently coloured contigs within components of a single colour. Figures 11b and 12b show the refined assembly graph where GraphBin2 has removed labels of unsupported vertices and corrected labels of inconsistent vertices. After GraphBin2 propagates labels to the remaining unlabelled vertices, the assembly graph will look as denoted in Figs. 11c and 12c. Finally, GraphBin2 will detect multi-labelled vertices that correspond to contigs that may belong to multiple species as shown by the black coloured vertices in Figs. 11d and 12d.

### Implementation, running time and memory usage

The source code for the experiments was implemented using Python 3.7.3 and run on a Darwin system with macOS Mojave 10.14.6, 16 GB memory and Intel Core i7 CPU @ 2.8 GHz with 4 CPU cores. In our experiments, we restrict the depth of the breadth-first-search in Steps 2-3 to be 5 to speed up GraphBin2. Moreover, we have set the parameter $\alpha =1.5$ by default for GraphBin2. Furthermore, the process of inferring multi-labelled vertices was performed in parallel using multithreading (set to 8 threads by default in GraphBin2).

The running times (wall time) and the peak memory used by the assemblers to assemble all the datasets, and the initial binning tools (CONCOCT, MaxBin2 and SolidBin) and GraphBin2 were recorded. All the running times and memory usage can be found in Section 4 of Additional file 1.

## Conclusion

In this paper we presented a novel algorithm, GraphBin2, that incorporates the coverage information into the assembly graph as an improvement of GraphBin [24]. While GraphBin uses only the topology of the assembly graph to refine and propagate labels, GraphBin2 makes use of the coverage information on vertices to perform label propagation. Moreover, GraphBin2 uses an improved label propagation algorithm that takes into consideration the distance and coverage of neighbouring contigs, compared to the label propagation algorithm used in GraphBin. Furthermore, GraphBin2 enables the detection of contigs that may belong to multiple species. The performance of GraphBin2 was evaluated against its predecessor and three other contig-binning tools on top of contigs obtained from short-reads assembled using metaSPAdes [27] and SGA [32] which represent the two assembly paradigms; de Bruijn graphs and overlap-layout-consensus (string graphs). The results showed that GraphBin2 achieves the best binning performance in both simulated and real datasets. Moreover, GraphBin2 shows the potential to infer contigs shared by multiple species. We have experimentally shown that GraphBin2 could be in principle applied to long-read assemblies. In the future, we intend to extend the capabilities of GraphBin2 to explore the avenues at improving the detection of contigs shared by multiple species, detection of misassemblies, and further extend towards binning long reads directly using read-overlap graphs.

## Supplementary Information

**Additional file 1.** Supplementary data including further details on datasets, commands used to run the tools, results of SGA assemblies and resource usage of the tools.

## Footnotes

1 In GraphBin, a vertex *i* is denoted as an *ambiguous* vertex if at least one of its closest labelled vertices has a label that is different than the label of the vertex *i*. An *ambiguous* vertex in GraphBin may be *supported* (in GraphBin2) by another vertex of the same label if they are directly connected or connected through a path consisting of only unlabelled vertices. An *unsupported* vertex in GraphBin2 is always *ambiguous* in GraphBin.
