# Supplementary material for: Improving metagenomic binning results with overlapped bins using assembly graphs
Source: Algorithms Mol Biol. 2021 May 4;16:3. doi: 10.1186/s13015-021-00185-6 (PMC8097841; doi:10.1186/s13015-021-00185-6)
Supplement: Supplementary file 1 — Additional file 1. Supplementary data including further details on datasets, commands used to run the tools, results of SGA assemblies and resource usage of the tools. [file 13015_2021_185_MOESM1_ESM.pdf]

Supplementary Material for "Improving Metagenomic Binning Results with Overlapped Bins Using Assembly Graphs"

1 Datasets

Supplementary Table 1 summarises the information on the datasets used for the experiments including read length, number of reads, number of contigs, mean contig length and the number of species identified for the ground truth. Paired-end reads were simulated for the **Sim-5G**, **Sim-10G**, **Sim-20G** and **50G-SR** datasets using the tool InSilicoSeq [2] modelling a MiSeq instrument with 300bp mean read length. The **Sharon** [7] datasets consisted of Illumina HiSeq 2000 reads with 100bp mean read length. The **Lake Water** [5] dataset consisted of Illumina MiSeq reads with 300bp mean read length. The **100G-LR** [10] dataset consisted of simulated PacBio reads with 8,000bp mean read length.

Dataset	Assembler	Read length (bp)	Number of paired end reads	Total number of non-isolated contigs	Mean contig length (bp)	Number of species in ground truth
Sim-5G	metaSPAdes	300	2,000,000	516	51,723	5
	SGA	300	2,000,000	18,192	1,675	5
Sim-10G	metaSPAdes	300	6,999,998	900	47,279	10
	SGA	300	6,999,998	32,389	1,300	10
Sim-20G	metaSPAdes	300	15,000,001	1,404	48,021	20
	SGA	300	15,000,001	72,791	873	20
Sharon-1 [7]	metaSPAdes	100	14,869,863	371	17,144	12
	SGA	100	14,869,863	766	3,034	12
Sharon-All [7]	metaSPAdes	100	135,493,567	2,730	7,689	12
	SGA	100	135,493,567	20,942	1,547	12
50G-SR	metaSPAdes	300	20,730,313	4,159	37,027	50
Lake Water [5]	metaSPAdes	300	4,627,091	96,880	1,020	57
100G-LR [10]	metaFlye	8,000	3,754,639	958	2,538	100

Supplementary Table 1: Information on the datasets used for the experiments.

Supplementary Table 2 denotes the details about the simulated short-read datasets including the species present, their genome sizes, sequencing coverage values and abundance values.

Dataset	Species present	Genome size	Coverage	Abundance
Sim-5G	Acetobacter pasteurianus	2.9 Mb	115×	28%
	Aeromonas veronii	4.6 Mb	72×	28%
	Amycolatopsis mediterranei	10.4 Mb	26×	22%
	Arthrobacter arilaitensis	3.9 Mb	41×	13%
	Azorhizobium caulinodans	5.4 Mb	20×	9%
Sim-10G	Acetobacter pasteurianus	2.9 Mb	357×	25%
	Aeromonas veronii	4.6 Mb	225×	25%
	Amycolatopsis mediterranei	10.4 Mb	80×	20%
	Arthrobacter arilaitensis	3.9 Mb	128×	12%
	Azorhizobium caulinodans	5.4 Mb	62×	8%
	Bacillus cereus	5.3 Mb	58×	7%
	Bdellovibrio bacteriovorus	3.8 Mb	11×	1%
	Bifidobacterium adolescentis	2.1 Mb	20×	1%
	Brachyspira intermedia	3.4 Mb	11×	1%
	Campylobacter jejuni	1.7 Mb	21×	1%
Sim-20G	Acetobacter pasteurianus	2.9 Mb	705×	23%
	Aeromonas veronii	4.6 Mb	445×	23%
	Amycolatopsis mediterranei	10.4 Mb	157×	18%
	Arthrobacter arilaitensis	3.9 Mb	253×	11%
	Azorhizobium caulinodans	5.4 Mb	123×	7%
	Bacillus cereus	5.3 Mb	114×	7%
	Bdellovibrio bacteriovorus	3.8 Mb	22×	1%
	Bifidobacterium adolescentis	2.1 Mb	40×	1%
	Brachyspira intermedia	3.4 Mb	21×	1%
	Campylobacter jejuni	1.7 Mb	41×	1%
	Candidatus Pelagibacter ubique	1.3 Mb	54×	1%
	Chlamydia trachomatis	1.1 Mb	64×	1%
	Clostridium acetobutylicum	4.0 Mb	18×	1%
	Corynebacterium diphtheriae	2.5 Mb	28×	1%
	Cyanobacterium UCYN	1.5 Mb	47×	1%
	Desulfovibrio vulgaris	3.6 Mb	20×	1%
	Ehrlichia ruminantium	1.5 Mb	47×	1%
	Enterococcus faecium	3.0 Mb	24×	1%
	Erysipelothrix rhusiopathiae	1.8 Mb	39×	1%
	Escherichia coli	5.0 Mb	14×	1%
50G-SR	Acetobacter pasteurianus	2.9 Mb	773×	4%
	Aeromonas veronii	4.6 Mb	493×	3%
	Amycolatopsis mediterranei	10.4 Mb	175×	2%
	Arthrobacter arilaitensis	3.9 Mb	281×	2%
	Azorhizobium caulinodans	5.4 Mb	136×	2%
	Bacillus cereus	5.3 Mb	126×	2%
	Bacillus thuringiensis	5.4 Mb	35×	2%
	Bdellovibrio bacteriovorus	3.8 Mb	25×	2%
	Bifidobacterium adolescentis	2.1 Mb	44×	2%
	Bifidobacterium animalis	2.0 Mb	48×	2%
	Brachyspira intermedia	3.4 Mb	23×	2%
	Campylobacter jejuni	1.7 Mb	47×	2%
	Candidatus Pelagibacter ubique	1.3 Mb	59×	2%
	Candidatus Phytoplasma mali	0.6 Mb	129×	2%
	Candidatus Sulcia muelleri	0.3 Mb	279×	2%
	Chlamydia psittaci	1.2 Mb	66×	2%
	Chlamydia trachomatis	1.1 Mb	74×	2%
	Clostridium acetobutylicum	4.0 Mb	20×	2%
	Clostridium botulinum	2.8 Mb	28×	2%
	Clostridium tetani	2.8 Mb	28×	2%
	Clostridium thermocellum	3.9 Mb	20×	2%
	Corynebacterium diphtheriae	2.5 Mb	31×	2%
	Corynebacterium pseudotuberculosis	2.4 Mb	33×	2%
	Corynebacterium ulcerans	2.5 Mb	31×	2%
	Cyanobacterium UCYN	1.5 Mb	54×	2%
	Cyanothece sp	6.2 Mb	13×	2%
	Desulfovibrio vulgaris	3.6 Mb	22×	2%
	Ehrlichia ruminantium	1.5 Mb	52×	2%
	Enterococcus faecium	3.0 Mb	26×	2%
	Erysipelothrix rhusiopathiae	1.8 Mb	43×	2%
	Escherichia coli	5.0 Mb	16×	2%
	Fervidicoccus fontis	1.3 Mb	59×	2%
	Fibrobacter succinogenes	3.9 Mb	20×	2%
	Flavobacterium branchiophilum	3.6 Mb	22×	2%
	Francisella novicida	1.9 Mb	41×	2%
	Francisella tularensis	1.9 Mb	41×	2%
	Fusobacterium nucleatum	2.2 Mb	36×	2%
	Gardnerella vaginalis	1.8 Mb	45×	2%
	Granulicella tundricola	4.4 Mb	18×	2%
	Haemophilus influenzae	1.9 Mb	41×	2%
	Haemophilus parainfluenzae	2.1 Mb	37×	2%
	Haemophilus somnus	2.3 Mb	34×	2%
	Halobacterium sp. NRC-1	2.0 Mb	38×	2%
	Halothiobacillus neapolitanus	2.6 Mb	30×	2%
	Helicobacter pylori	1.6 Mb	49×	2%
	Hyphomicrobium sp. MC1	4.9 Mb	16×	2%
	Ignavibacterium album	3.7 Mb	21×	2%
	Klebsiella oxytoc	6.1 Mb	13×	2%
	Krokinobacter sp	3.4 Mb	23×	2%
	Lactobacillus brevis	2.3 Mb	34×	2%

Supplementary Table 2: Details about the simulated short-read datasets.

2 Commands Used

2.1 Assembly Tools

metaSPAdes

spades --meta -1 Reads_1.fastq -2 Reads_2.fastq -o /path/output_path -t 20

SGA

sga preprocess -o reads.fastq --pe-mode 1 Reads_1.fastq Reads_2.fastq
sga index -a ropebwt -t 16 --no-reverse reads.fastq
sga correct -k 41 --learn -t 16 -o reads.k41.fastq reads.fastq
sga index -a ropebwt -t 16 reads.k41.fastq
sga filter -x 2 -t 16 reads.k41.fastq
sga fm-merge -m 45 -t 16 reads.k41.filter.pass.fa
sga index -t 16 reads.k41.filter.pass.merged.fa
sga overlap -m 55 -t 16 reads.k41.filter.pass.merged.fa
sga assemble -m 95 reads.k41.filter.pass.merged.asqg.gz

metaFlye

flye --meta --pacbio-raw reads.fasta --genome-size estimated_metagenome_size --out-dir /output_path --threads 16

2.2 Binning and Refinement Tools

CONCOCT

cut_up_fasta.py contigs.fasta -c 10000 -o 0 --merge_last -b contigs_10K.bed > contigs_10K.fa
concoct_coverage_table.py contigs_10K.bed aln-pe.sorted.bam > coverage_table.tsv
concoct --composition_file contigs_10K.fa --coverage_file coverage_table.tsv -b /output_path -t 8
merge_cutup_clustering.py /output_path/clustering_gt1000.csv > /output_path/clustering_merged.csv
extract_fasta_bins.py contigs.fasta /output_path/clustering_merged.csv --output_path /output_path/fasta_bins

MaxBin2

perl MaxBin-2.2.5/run_MaxBin.pl -contig contigs.fasta -abund abundance.abund -out /output_path

SolidBin

python scripts/gen_kmer.py contig.fasta 1000 4
sh gen_cov.sh
python SolidBin.py --contig_file contigs.fasta --composition_profiles kmer_4.csv --coverage_profiles cov_inputtableR.tsv
--output /output_path/result.tsv --log /output_path/log.txt --use_sfs

GraphBin

./graphbin --graph assembly_graph_with_scaffolds.gfa --paths contigs.paths --binned initial_contig_bins.csv --output /output_path --assembler spades

3 Results of SGA Assemblies

3.1 Binning Results

Supplementary Figures 1, 2 and 3 demonstrate the results of CONCOCT [1], MaxBin2 [11] and SolidBin [9], respectively with GraphBin [4] and GraphBin2 on top of the initial binning results for the SGA [8] assemblies.

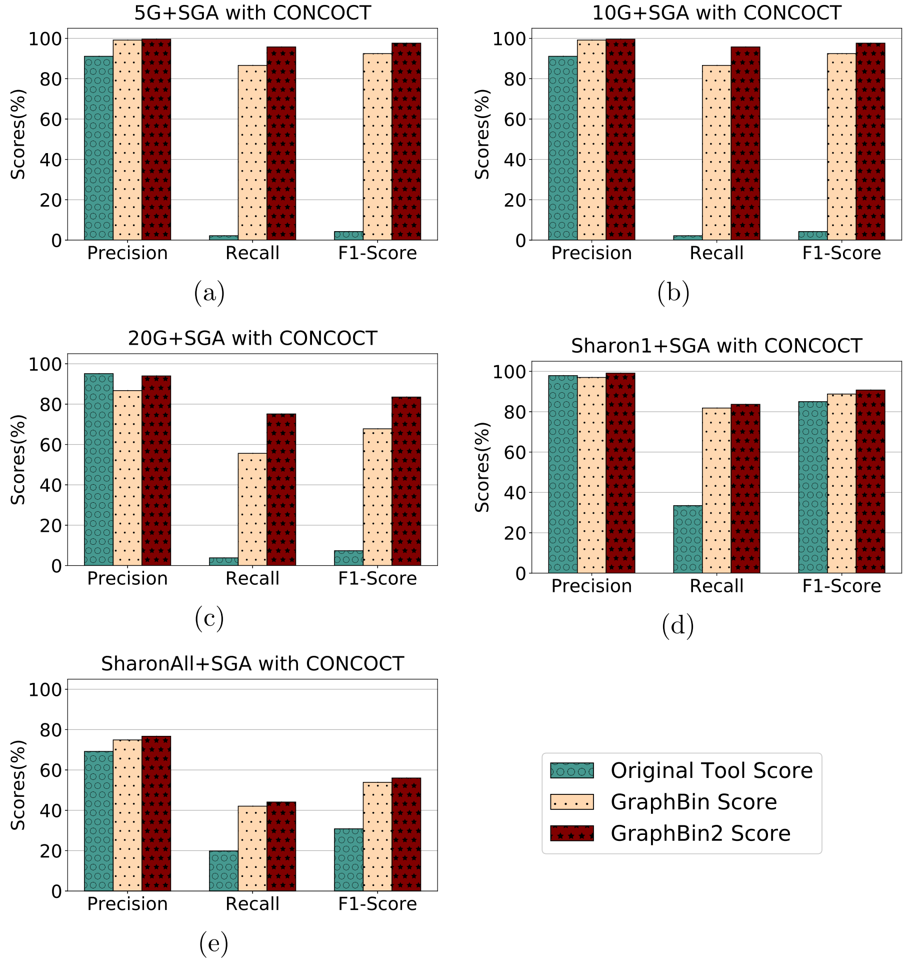

Supplementary Figure 1: Comparison of binning results of CONCOCT [1], GraphBin [4] and GraphBin2 (on top of CONCOCT results) using assembly graphs built by SGA [8].

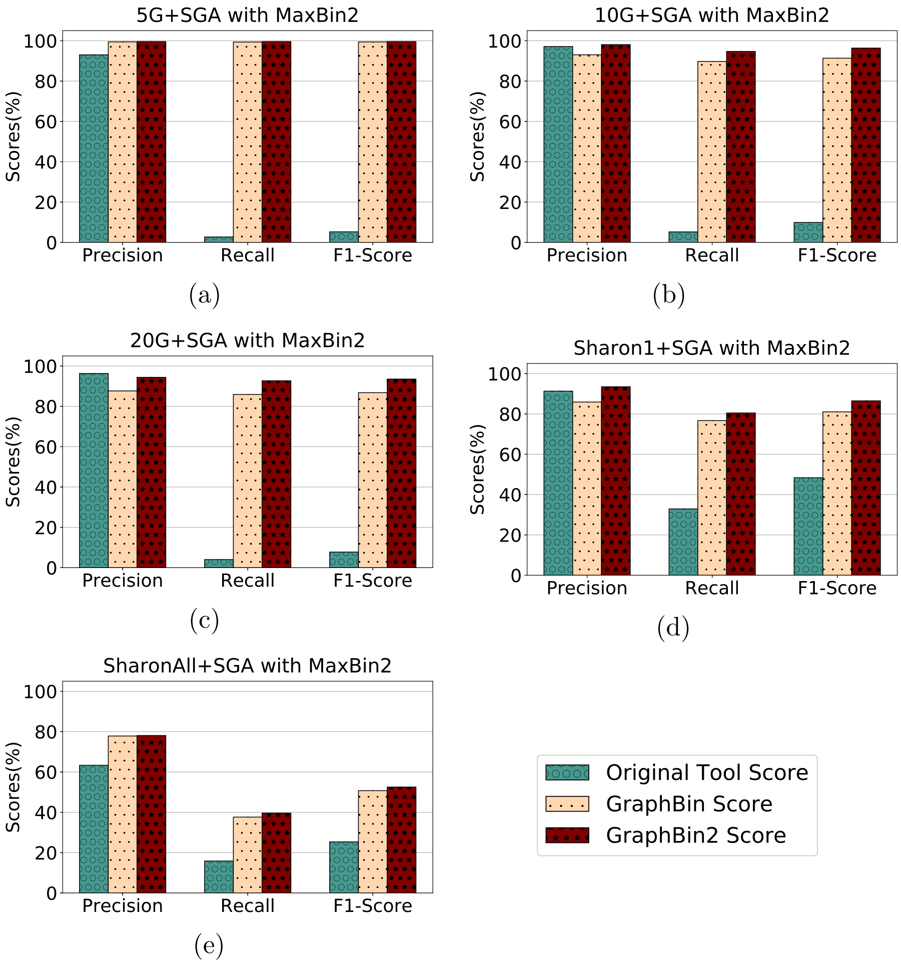

Supplementary Figure 2: Comparison of binning results of MaxBin2 [11], GraphBin [4] and GraphBin2 (on top of MaxBin2 results) using assembly graphs built by SGA [8].

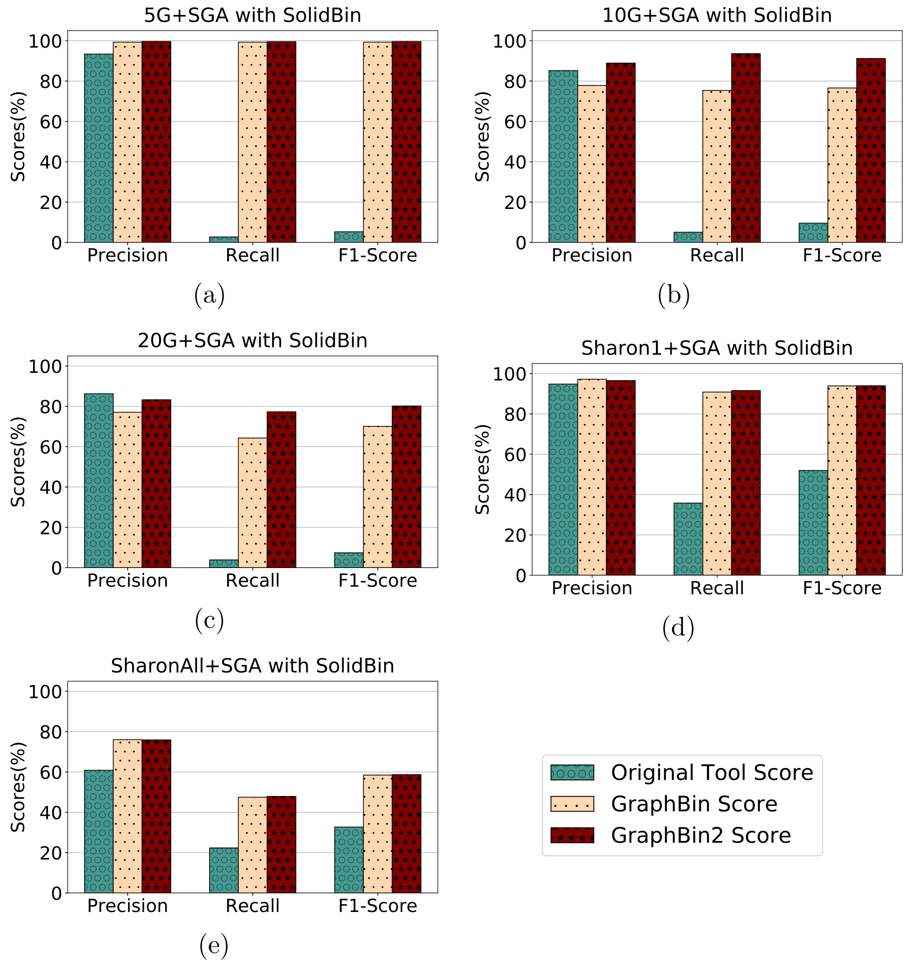

Supplementary Figure 3: Comparison of binning results of SolidBin [9], GraphBin [4] and GraphBin2 (on top of SolidBin results) using assembly graphs built by SGA [8].

3.2 Multi-Labelled Inference Results

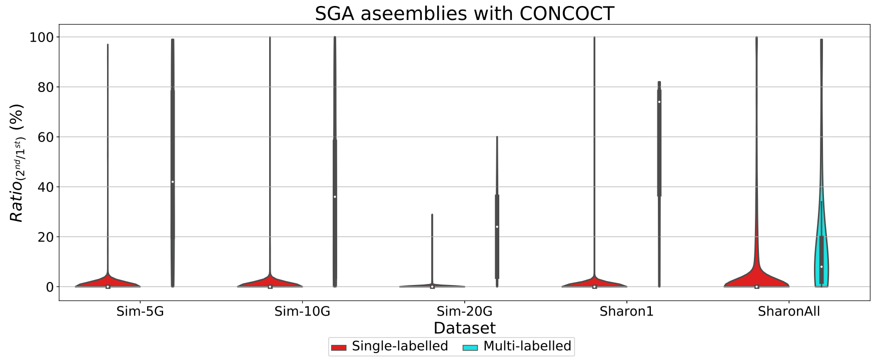

(a)

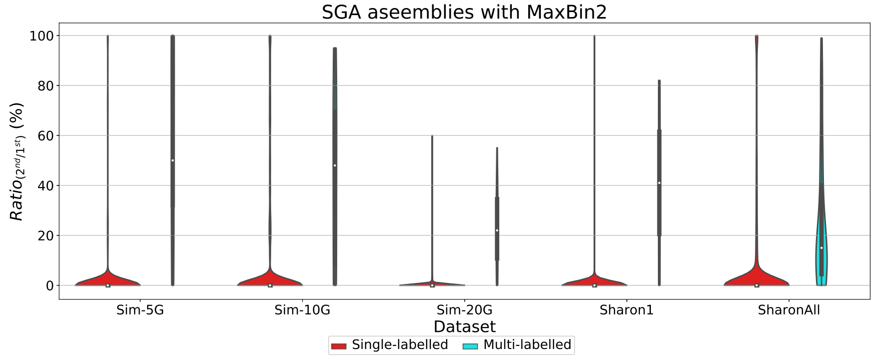

(b)

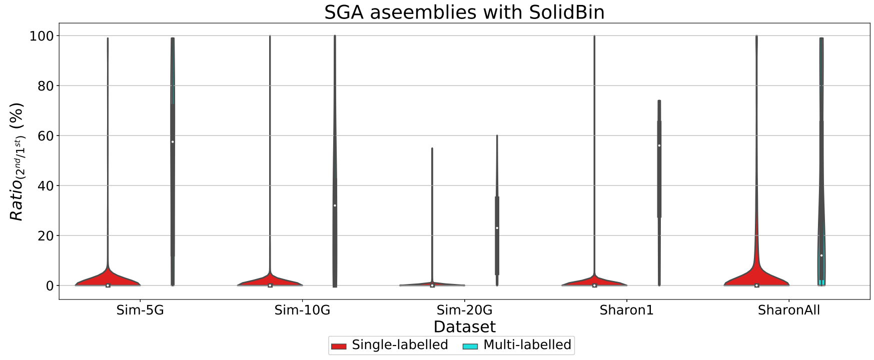

(c)

Supplementary Figure 4: Violin plots for the ratio $Ratio_{(2^{nd}/1^{st})}$ of the single and multi-labelled inference results using GraphBin2 on top of (a) CONCOCT [1], (b) MaxBin2 [11] and (c) SolidBin [9] results for the SGA assemblies.

Dataset	With CONCOCT result	With MaxBin2 result	With SolidBin result
Sim-5G	31	6	8
Sim-10G	81	9	2
Sim-20G	156	15	11
Sharon1	6	2	2
SharonAll	40	37	17

Supplementary Table 3: The number of multi-labelled contigs identified by GraphBin2 for the SGA [8] assemblies using the initial binning result of each binning tool.

4 Running Time and Memory Usage

The running times and the peak memory used by the metaSPAdes [6] and metaFlye [3] for assembly, the initial binning tools and GraphBin2 to bin all the datasets can be found in Supplementary Tables 4, 5 and 6.

Dataset	Assembly	Running time (CPU time)	Peak memory usage
Sim-5G	metaSPAdes	2h 42m 5s	7.78 GB
Sim-10G	metaSPAdes	16h 07m 58s	24.60 GB
Sim-20G	metaSPAdes	44h 54m 17s	54.33 GB
Sharon1 [7]	metaSPAdes	4h 27m 29s	1.66 GB
SharonAll [7]	metaSPAdes	78h 57m 24s	199.93 GB
50G-SR	metaSPAdes	77h 30m 6s	70.67 GB
Lake Water [5]	metaSPAdes	17h 21m 52s	51.79 GB
100G-LR [10]	metaFlye	129h 21m 58s	299.11 GB

Supplementary Table 4: Running times (CPU time) and peak memory usage to assemble each dataset. *s* denotes seconds, *m* denotes minutes, *h* denotes hours and *GB* denotes gigabytes.

CONCOCT, MaxBin2 and GraphBin2 were executed with 8 threads and SolidBin was executed with a single thread. The running times for CONCOCT and SolidBin only include the times taken to run the main software, excluding the times taken to build the composition and coverage profile files.

GraphBin2 took less than 12 minutes and less than 165 MB of memory to complete executing the **Sharon-All** dataset with 8 threads. Moreover, the highest running time and memory usage has been recorded for the metaSPAdes assembly of the **LakeWater** [5] dataset as it consisted of the most complex assembly graph with the most number of contigs.

Dataset	Assembly	Tool	Running time	Peak memory usage
Sim-5G	metaSPAdes	CONCOCT	29s	172 MB
		GraphBin2 with CONCOCT	1s	35 MB
		MaxBin2	12s	2,389 MB
		GraphBin2 with MaxBin2	1s	36 MB
		SolidBin	3s	155 MB
		GraphBin2 with SolidBin	1s	36 MB
	SGA	CONCOCT	20s	169 MB
		GraphBin2 with CONCOCT	3m 58s	127 MB
		MaxBin2	15s	394 MB
		GraphBin2 with MaxBin2	3m 12s	124 MB
		SolidBin	3m 1s	794 MB
		GraphBin2 with SolidBin	3m 54s	124 MB
Sim-10G	metaSPAdes	CONCOCT	25s	175 MB
		GraphBin2 with CONCOCT	2s	40 MB
		MaxBin2	20s	2,859 MB
		GraphBin2 with MaxBin2	2s	41 MB
		SolidBin	3s	164 MB
		GraphBin2 with SolidBin	2s	41 MB
	SGA	CONCOCT	14s	204 MB
		GraphBin2 with CONCOCT	4m 33s	101 MB
		MaxBin2	28s	285 MB
		GraphBin2 with MaxBin2	5m	101 MB
		SolidBin	8m 25s	1,423 MB
		GraphBin2 with SolidBin	5m 2s	101 MB
Sim-20G	metaSPAdes	CONCOCT	41s	193 MB
		GraphBin2 with CONCOCT	3s	44 MB
		MaxBin2	32s	2,854 MB
		GraphBin2 with MaxBin2	3s	44 MB
		SolidBin	4s	193 MB
		GraphBin2 with SolidBin	5s	45 MB
	SGA	CONCOCT	25s	211 MB
		GraphBin2 with CONCOCT	28m 45s	194 MB
		MaxBin2	49s	364 MB
		GraphBin2 with MaxBin2	29m 40s	192 MB
		SolidBin	18m 47s	2,064 MB
		GraphBin2 with SolidBin	29m 54s	193 MB
50G-SR	metaSPAdes	CONCOCT	1m 35s	237 MB
		GraphBin2 with CONCOCT	19s	75 MB
		MaxBin2	1m 33 s	3,978 MB
		GraphBin2 with MaxBin2	33s	77 MB
		SolidBin	13s	500 MB
		GraphBin2 with SolidBin	21s	75 MB

Supplementary Table 5: Running times (wall time) and peak memory usage for binning using each tool for the simulated short-read datasets. *s* denotes seconds, *m* denotes minutes and *MB* denotes megabytes.

Dataset	Assembly	Tool	Running time	Peak memory usage
Sharon1	metaSPAdes	CONCOCT	12s	166 MB
		GraphBin2 with CONCOCT	4s	45 MB
		MaxBin2	9s	1,389 MB
		GraphBin2 with MaxBin2	5s	45 MB
		SolidBin	6s	290 MB
		GraphBin2 with SolidBin	5s	45 MB
	SGA	CONCOCT	20s	172 MB
		GraphBin2 with CONCOCT	3s	33 MB
		MaxBin2	12s	203 MB
		GraphBin2 with MaxBin2	3s	33 MB
		SolidBin	15s	654 MB
		GraphBin2 with SolidBin	3s	33 MB
SharonAll	metaSPAdes	CONCOCT	1m 8s	189 MB
		GraphBin2 with CONCOCT	9m 54s	137 MB
		MaxBin2	30s	1,378 MB
		GraphBin2 with MaxBin2	10m 50s	163 MB
		SolidBin	2m 7s	1,416 MB
		GraphBin2 with SolidBin	11m 12s	163 MB
	SGA	CONCOCT	1m 46s	201 MB
		GraphBin2 with CONCOCT	1m 13s	50 MB
		MaxBin2	28s	241 MB
		GraphBin2 with MaxBin2	1m 21s	50 MB
		SolidBin	2m 51s	2,612 MB
		GraphBin2 with SolidBin	1m 15s	50 MB
Lake Water	metaSPAdes	CONCOCT	22m 2s	807 MB
		GraphBin2 with CONCOCT	58m 42s	855 MB
		MaxBin2	23m 27s	1,004 MB
		GraphBin2 with MaxBin2	55m 17s	862 MB
		SolidBin*	N/A*	N/A*
		GraphBin2 with SolidBin*	N/A*	N/A*
100G-LR	metaFlye	CONCOCT	3m 7s	399 MB
		GraphBin2 with CONCOCT	9s	54 MB
		MaxBin2	4m 8s	3,976 MB
		GraphBin2 with MaxBin2	4s	57 MB
		SolidBin	14m 59s	4,840 MB
		GraphBin2 with SolidBin	4s	57 MB

Supplementary Table 6: Running times (wall time) and peak memory usage for binning using each tool for the remaining real and long-read datasets. s denotes seconds, m denotes minutes and MB denotes megabytes.

* SolidBin [9] could not be run on the Lake Water dataset due to insufficient memory.

References

[1] Johannes Alneberg, Brynjar Smári Bjarnason, Ino de Bruijn, Melanie Schirmer, Joshua Quick, Umer Z. Ijaz, Leo Lahti, Nicholas J. Loman, Anders F. Andersson, and Christopher Quince. Binning metagenomic contigs by coverage and composition. *Nature Methods*, 11:1144–1146, Sep 2014.

[2] Hadrien Gourlé, Oskar Karlsson-Lindsjö, Juliette Hayer, and Erik Bongcam-Rudloff. Simulating Illumina metagenomic data with InSilicoSeq. *Bioinformatics*, 35(3):521–522, 07 2018.

[3] Mikhail Kolmogorov, Derek M. Bickhart, Bahar Behsaz, Alexey Gurevich, Mikhail Rayko, Sung Bong Shin, Kristen Kuhn, Jeffrey Yuan, Evgeny Polevikov, Timothy P. L. Smith, and Pavel A. Pevzner. metaflye: scalable long-read metagenome assembly using repeat graphs. *Nature Methods*, 17(11):1103–1110, Nov 2020.

[4] Vijini Mallawaarachchi, Anuradha Wickramarachchi, and Yu Lin. GraphBin: Refined binning of metagenomic contigs using assembly graphs. *Bioinformatics*, 03 2020. btaa180.

[5] Maliheh Mehrshad, Michaela M. Salcher, Yusuke Okazaki, Shin-ichi Nakano, Karel Šimek, Adrian-Stefan Andrei, and Rohit Ghai. Hidden in plain sight—highly abundant and diverse planktonic freshwater chloroflexi. *Microbiome*, 6(1):176, Oct 2018.

[6] Sergey Nurk, Dmitry Meleshko, Anton Korobeynikov, and Pavel A. Pevzner. metaSPAdes: a new versatile metagenomic assembler. *Genome Research*, 27(5):824–834, 2017.

[7] Itai Sharon, Michael J. Morowitz, Brian C. Thomas, Elizabeth K. Costello, David A. Relman, and Jillian F. Banfield. Time series community genomics analysis reveals rapid shifts in bacterial species, strains, and phage during infant gut colonization. *Genome Research*, 23(1):111–120, 2013.

[8] Jared T. Simpson and Richard Durbin. Efficient de novo assembly of large genomes using compressed data structures. *Genome Research*, 22(3):549–556, 2012.

[9] Ziye Wang, Zhengyang Wang, Yang Young Lu, Fengzhu Sun, and Shanfeng Zhu. SolidBin: improving metagenome binning with semi-supervised normalized cut. *Bioinformatics*, 35(21):4229–4238, 04 2019.

[10] Anuradha Wickramarachchi, Vijini Mallawaarachchi, Vaibhav Rajan, and Yu Lin. MetaBCC-LR: metagenomics binning by coverage and composition for long reads. *Bioinformatics*, 36(Supplement_1):i3–i11, 07 2020.

[11] Yu-Wei Wu, Blake A. Simmons, and Steven W. Singer. MaxBin 2.0: an automated binning algorithm to recover genomes from multiple metagenomic datasets. *Bioinformatics*, 32(4):605–607, Oct 2015.
